# Student nurses’ knowledge acquisition on oral medication administration: comparison of lecture demonstration vs. video demonstration

**DOI:** 10.1186/s12912-020-00527-6

**Published:** 2021-01-06

**Authors:** Ravilal Devananda Udeshika Priyadarshani Sugathapala, Mattaka Gamage Ruwini Chandrika

**Affiliations:** Department of Nursing and Midwifery, Faculty of Allied Health Sciences, General Sir John Kotelawala Defence University, Ratmalana, Sri Lanka

**Keywords:** Nursing students, Oral medication administration, Knowledge acquisition

## Abstract

**Background:**

Oral Medication administration is one of the paramount nursing procedures, where nurses must pay their utmost commitment. The vital aims are to reduce medication errors and ensure patient safety. The objectives of this study were to evaluate whether the nursing students could learn and retain the basic guidelines for oral medication administration when they are taught using a video-assisted teaching method compared with the lecture-demonstration method and to assess the students’ attitudes towards the two types of teaching methods.

**Methods:**

This study was conducted as a quasi-experimental study with a pre and post-test design. Forty-five students in the first year of the bachelor’s degree in nursing programme participated. All the participants completed a self- administered questionnaire, including socio demographic data and questions of oral medication administration. Subsequently, participants were randomly assigned to two groups. Oral medication administration procedure was taught using two different teaching methods. Finally, the post-test knowledge scores of both groups were assessed and analysed using the paired-sample t-test.

**Results:**

The results revealed that there was no significant difference in terms of age, gender and type of residence of students in the two groups. When comparing the pre-test mean score and post-test mean score using paired sample t-test, there was a statistically significant difference in both video demonstration group (t = − 4.533, *p* < 0.001) and lecture-demonstration group (t = − 4.208, *p* < 0.001). Almost all the students obtained good knowledge scores regardless of the method used in teaching oral medication administration. However, when comparing post-test scores of both groups using an independent sample t-test, it was identified that there was no significant difference between the two groups. Therefore, it was difficult to identify which method was effective than the other. According to the student feedback obtained at the end of the study, 67% of them preferred to have more video demonstrations in their skills classes.

**Conclusion:**

The results of this study suggested that oral medication administration can be effectively taught using lecture-demonstration and video-demonstration teaching methods.

**Supplementary Information:**

The online version contains supplementary material available at 10.1186/s12912-020-00527-6.

## Background

To maintain standard nursing practices, the nursing staff must incorporate clinical competencies within themselves. Furthermore, high proficiency in nursing care increases the safety of the patient within the clinical context. Therefore, nursing students should be guided based on a practice-oriented way, and it will help in building up confidence and empower them to actively participate in the care of patients [1]. Obtaining the required clinical competency level is mandatory not only for practice as a professional but also it will enhance the problem solving and reasoning capabilities; inculcating dedication to the service, and ultimately increasing the quality patient care. Technical knowledge has created an impact on the teaching and learning process. Telecommunication allows the learners to collect more details to resolve the issues and to be competent enough in clinical practice [2].

Pre-registration nursing education was based on a 3 year diploma level nursing program in schools of nursing that are attached to the Ministry of Health, Sri Lanka. It was proposed that the entire nursing education should be upgraded to a degree level. As a result, the University Grants Commission (UGC) in Sri Lanka has established four-year degree programs leading to BSc (Honours) in Nursing in five universities. In 2012, General Sir John Kotelawala Defence University, Sri Lanka established the Faculty of Allied Health Sciences (FAHS), and FAHS caters civil nursing undergraduates by offering a four-year BSc (Honours) in Nursing degree programme.

The nursing curriculum encompasses scientific and humanistic foundation for nursing practice and enables students to develop competencies related to prevention, maintenance, cure, rehabilitation and promotion of health. The degree program is fulltime, and module-based with 126 credits allocated to modules. Out of 126 total credits, 59 credits are allocated for teaching theory and 54 credits are allocated to hospital-based clinical training. Before students are placed in-hospital training, they are supposed to learn and practice nursing procedures within the skills lab. The skills lab practical demonstrations are taught from the beginning of the first year to the final year. These range from basic nursing procedures to advanced nursing procedures.

Administrating oral medication to patients is one of the essential procedures supposed to be taught during the first year second semester. It is crucial to have excellent decision-making skills and clinical judgment for a nurse, and the nurse is responsible for making sense of medication administration and its implications for patient safety. Maintaining quality and safety care is essential to meet the needs of the patient [3].

Alterations of the teaching methodologies have become a challenge at present due to the rapid transformation of the nursing curriculum. Since students have different learning styles, the teachers should deliver the lessons according to the learning styles of the students. Therefore, teachers should implement different teaching methods and techniques other than the conventional talk and chalk method to teach different skills. A lecture-demonstration is a method where the instructor performs the procedure while explaining every step of the procedure with the rationale behind it. By using this method, the students will be able to develop essential qualities in learning science ─ independent thinking, power of observation and reasoning. Learning of nursing psychomotor skills has traditionally been associated with lecture- demonstrations in the skills laboratories. The skill laboratories help ensure all students acquire the necessary techniques before practicing on real patients [4]. Procedural videos can provide a valued teaching tool that allows better visualisation of procedural steps, offer media-rich audio and visual stimulation covering a wider spectrum of learning styles or preferences which allow students to review nursing procedures before, during, or after the sessions, and they can help overcome shortage of time for onsite learning. In terms of skills demonstrations, video-based education is used when demonstrations are impossible to be undertaken. Further, the students have the opportunity to listen to the voice and watch diagrams and figures in video-based education [5].

Clinical learning is an important aspect of nursing education. A clinical demonstration is a method of presentation of skills which shows how a particular procedure is performed in the clinical setting. Students’ exposure to clinical learning environment is one of the most pivotal factors that affects the teaching-learning process in clinical settings [6].

Opportunities for teachers to provoke the thinking and practical application of students for a particular subject is connected to new teaching methods and ideologies: this method will lead students to become creative thinkers and inventors. Identifying a better teaching method in oral medication administration is necessary for the teacher to enhance students’ performance and that can contribute more in the field of treatment skills. It will result in positive outcomes in future health indicators and quality of patient care.

Though there are many studies done in this area in other countries, few studies were found in Sri Lanka. The present study aims to determine the effectiveness of video demonstration in clinical skills education of medication administration for undergraduate nursing students compared to the lecture-demonstration method.

## Methods

### Study design

A quasi-experimental study with a pre and post-test design was utilised to determine whether the nursing students could learn and retain the theoretical knowledge of oral medication administration when taught using a video demonstration compared with the conventional demonstration method. Students’ attitudes regarding the two teaching methods were also assessed at the end of the study. A questionnaire and a video clip were developed specifically for these purposes.

### Participants and setting

Considering the effects of the dependant variable of this study, only the first year BSc Nursing undergraduates of General Sir John Kotelawala Defence University were recruited as participants for the study. These students have never been exposed to oral medication administration procedure before. The study was conducted in 2019 at the Faculty of Allied Health Sciences, General Sir John Kotelawala Defence University.

### Hypothesis

The hypothesis generated for this study was that the nursing students who participated in the video demonstration would have increased knowledge score related to oral medication administration than the nursing students who have experienced the lecture demonstration in the skills lab.

### Sample size

The sample included all the nursing students enrolled in the first year of BSc in Nursing programme. Among the 45 first-year BSc nursing students, 22 students were randomly selected and assigned into video demonstration group, and the lecture-demonstration group consisted of 23 students.

### Data collection tools and methods

Data collection tool was a self-administered questionnaire which consisted of three parts. Part I covered demographic information, Part II questions on theoritical aspects on oral medication administration and Part III consisted of questions on attitudes regarding the two types of teaching methods. Knowledge acquisition on oral medication administration was measured using Part II which consisted of 15 Single Best choice Answer (SBA) questions developed by the researchers. These questions were classified into four subsections as patient safety (07 SBAs), preparation of medications (02 SBAs), special considerations (04 SBAs) and procedural steps (02 SBAs). Part III was also developed by the researchers using a Likert scale in order to assess the attitudes of the students towards the two different teaching methods. Part III was administered to all the study participants at the end of the data collection process. The self administered questionnaire was pre tested to ensure understanding of the items, wording and adequacy of response using 12 first year nursing undergraduates of a non state university and amended accordingly. Since, the same knowledge questionnaire was administered before and after teaching of oral medication administration as the pre-test and post-test, the test – retest reliability was assessed and the pearson correlation was significant (*p* = 0.001). An additional file shows the questionnaire in more detail (see Additional file 1).

The main objective of this study was to assess the effectiveness of video –demonstration method compared to the lecture demonstration method with regards to the knowledge acquisition of oral medication administration. Therefore, a video demonstration and a lecture demonstration were separately developed by the research team. Important priniciples underpinning the oral medication administration were included in both lecture and video demonstrations. Six rights of medication administration, i.e. right patient, right drug, right dose, right route, right time and right documentation and three checks of medication administration were included as patient safety measures. Assessment of the patient, preparation of environment and equipment, sequence of the procedure, prevention of cross infection, how to prepare the medication if it is a packaged tablet/capsule or a liquid medication, and proper waste disposal and cleaning and storing utensils after the procedure were incorpeoated as principles of medication administration.

The oral medication administration video clip was created using an adult manikin as the patient. The video recording was done in the skills lab setting using the same equipment which is used in the lecture-demonstration. The same lecturer did video dubbing who conducted the lecture-demonstration. The total video was 10 min. The video demonstration is the exact replication of the lecture – demonstration. The final version of the video clip was shared with two experienced clinical instructors in order to verify the accuracy of the performance and recording quality. In the lecture – demonstration, the teaching of correct steps of oral medication administration by the lecturer was adopted in the skills laboratory setting.

After completing the sociodemographic data form, the pre-test knowledge scores of both groups were assessed using Part II of the questionnaire. After that, the lecture-demonstration group (*n*=23) received the oral medication administration through the lecture-demonstration, whereas, the video demonstration group (*n*=22) was taught the oral medication administration procedure by the same lecturer using the video clip on a projected screen with speakers in the skills lab setting on the same day. Finally, the post-test and the attitude questions were administered to both groups on the 8th day after delivering lecture demonstration and video demonstration.

### Ethical considerations

This study was approved by the ethical review committee of the Faculty of Medicine, General Sir John Kotelawala Defence University with registration no. RP/2018/10, dated January 30, 2019. Furthermore, the permission to enrol undergraduates of KDU for this study was obtained from the Vice-Chancellor. All the enrolled students signed an informed consent containing clear information about the study, its purpose, and methods.

### Statistical analysis

Pre-test baseline characteristics for the experimental and control groups were examined using t-tests and χ^2^ tests. The paired t-test was used to compare the mean differences in knowledge based on the questionnaire within groups. The independent sample t-test was used to compare the pre-test and post-test self-confidence scores within groups. A difference was considered significant when the *p*-value was less than 0.05.

## Results

### Participants characteristics

Forty-five students participated in the study at a response rate of 100%. There were 13 (28.9%) male and 32 (71.1%) female students with a mean age of 21.24±0.86 years. The sociodemographic characteristics for the lecture-demonstration group and the video-demonstration group are shown in Table 1. According to the results of Fisher’s Exact Test, there was no statistically significant difference between the two groups in terms of gender, nationality, religion and type of residence.

**Table 1:** Sociodemographic characteristics of the study participants

Group	Video-assisted Teaching group (*N* = 22)	Lecture demonstration group (*N* = 23)
Age of students in years; mean (SD)	21.36 (0.85)	21.13 (0.87)
Gender, n (%)		
Male	06 (13.3)	07 (15.6)
Female	16 (35.6)	16 (35.6)
Nationality, n (%)		
Sinhala	21 (95.5)	21 (91.3)
Tamil	0 (0)	2 (8.7)
Muslim	01 (4.5)	0 (0)
Religion, n (%)		
Buddhist	21 (95.5)	19 (82.6)
Hindu	0 (0)	2 (8.7)
Christian	0 (0)	2 (8.7)
Islam	1 (4.5)	0 (0)
Residence, n (%)		
Home	5 (22.7)	2 (8.7)
University Hostel	0 (0)	2 (8.7)
Boarding House	17 (77.3)	19 (82.6)

### Knowledge scores on oral medication administration

The students answered 15 knowledge questions on oral medication administration and obtained the pre-test score. According to the results, the lecture demonstration group had a mean knowledge score of 47.20±14.08 out of 100, whereas, the video-assisted teaching group showed 48.38±16.92 of mean knowledge score. The pre-test scores of both groups were compared with the post-test scores, and the results are shown in Table 2. Based on the paired t-test result, there was a statistically significant difference between the pre-test and post-test scores of both video demonstration and lecture-demonstration method [(t = − 4.533, *p* < 0.001) and (t = − 4.208, *p* < 0.001)]. Almost all the students obtained good knowledge scores regardless of the method used in teaching oral medication administration (Table 2).

**Table 2:** Knowledge scores on oral medication administration

Knowledge scores	Video-assisted teaching group	Lecture demonstration group
Pre-test score	48.38 (SD =16.92)	47.20 (SD = 14.08)
Post-test score	65.90 (SD =17.06)	60.25 (SD =15.80)
“t”	−4.533	−4.208
Paired t test p	0.001	0.001

Furthermore, an independent sample t-test was performed for comparing the post-test results of the lecture-demonstration group and the video-assisted teaching group to identify whether there is a significant mean difference between the two groups. Post-test marks were normally distributed in the two groups, and equal variances assumed according to the Levene’s Test for Equality of Variances and gave a Sig. (2-tailed) = 0.254. Hence, it shows that there is no significant difference between the post-test marks of the two groups.

### Students responses to attitude questionnaire

Students were asked the question “Do you feel the videos were an enjoyable way to study?”. As per their response, 82% agreed with that statement. 66% of them agreed to incorporate videos into the skills demonstrations in the future. However, 82% agreed to learn skills classes as a demonstration in the skills lab. It was found that most of them were satisfied with a combination of lecture and video demonstration methods while learning skills practice in nursing (Table 3).

**Table 3:** Frequency of students responding to different attitude questions

Statements	Level of Agreement			
	Strongly Agree	Agree	Neutral	Disagree
I enjoy learning skills through video	05 (11.11)	32 (71.11)	04 (8.89)	04 (8.89)
I enjoy learning skills through demonstration by a lecturer in the skills lab	19 (42.22)	18 (40.00)	08 (17.78)	0 (0.00)
I felt prepared for the skills class after I watched the videos	12 (26.67)	16 (35.56)	15 (33.33)	02 (4.44)
I feel the videos and skills classes have prepared me for the skills in clinical practice	06 (13.33)	33 (73.33)	06 (13.33)	0 (0.00)
I would like videos to be used more in skills teaching	04 (8.89)	26 (57.78)	09 (20.00)	06 (13.33)
I feel I will use the videos to revise clinical skills in the future	09 (20.00)	30 (66.67)	06 (13.33)	0 (0.00)
I feel motivated to learn the skills through video	07 (15.56)	26 (57.78)	10 (22.22)	02 (4.44)

## Discussion

Teaching methods are based on principles and a variety of educational methods. They serve as suggestions, instructions, guidance and encouragement to learn. New methods and training materials are developing every day in research. A lecture-demonstration, a clinical demonstration, a video demonstration or combinations of these methods are used mostly. For better and quality patient care, the nurses should learn the nursing theories and clinical practice as they are updated with the time. Training programs are, therefore, essential in order to close the gap between theory and practice.

This study aimed to evaluate whether nursing students could learn and retain the theoretical aspects of oral medication administration when they were taught using a video demonstration compared to the conventional face-to-face teaching method. The findings of the study revealed that the students of lecture demonstration group increased the mean knowledge score from 47.20±14.08 to 60.25±15.80 and those learned through video demonstration has an improvement of mean knowledge score from 48.38±16.92 to 65.90 ±17.06. Both groups achieved considerable knowledge regarding medication administration after teaching the contents utilising different two types of teaching methods according to the paired sample t test results. Similar to the findings of the present study, another study was done to examine the effectiveness of video teaching (experimental group) over lecture-demonstration (control group) in increasing knowledge and skill of third-year nursing students on antenatal examination. There was a significant difference in the pre and post-test knowledge scores within experimental and control groups at 0.001 level of significance. It was noted that both groups were able to acquire a theoretical and practical understanding of antenatal examination [7].

Although, there was a significant improvement of knowledge on oral medication administration among both groups, we could not find a significant difference between post –test mean scores of the two groups, as per the results of independent sample t –test (*p* > 0.001). These results indicated that both lecture – demonstration and video-demonstration methods found equally effective for acquiring the theoretical aspects of oral medication administration. These findings were supported by a prospective study done in the University of London to assess the relative effectiveness of the two approaches of video demonstration and lecture-demonstration teaching methods for orthodontic auxiliary training. There was no significant difference between the teaching methods except for bracket positioning where video was slightly better (*P* < 0.05) [8]. Another study was done to compare two forms of teaching methods such as video and live lecture for education in clinical periodontology resulted that the live lecture group performed better than the video group during the in-depth post-test assessment and more students prefered video method. At the same time, most of the students prefered an ultimate combination of video and lecture also [9].

In contrast to the results of the present study, Devi (2019) reported that the traditional demonstration method was more effective than video demonstration in teaching obstetrical palpation [10].

A two-group quasi-experimental study was conducted on 165 baccalaureate nursing students to assess the effect of a video-based case study over a written case study. According to the findings, the video-based learners gained higher scores for knowledge testing, compared to the written case study group of learners. This study suggested that video-based education method could be used as an additional method to improve classroom teaching [11].

Most students viewed videos in an enjoyable way to study in the present study. Similarly, many students were of the opinion that the video and lecture demonstration methods motivated themselves for the preparation of skills practice at the actual clinical settings. Interestingly, 82% of students preferred lecture – demonstration method showing that they need to learn through observation and independent thinking. However, when considering the attitudes of the students they preferred both teaching methods and the reasons might be, they could learn the principles of oral medication administration through the lecture demonstration and they could use the video demonstration for the purpose of preview and review. These findings were supported by a randomized controlled trial with a pre-test and post-test design conducted among 71 nursing students. A video clip on how to perform urinary catheterization was developed, and the intervention group was able to download it on to their own mobile devices. All the students participated in the lecture on urinary catheterization. Results indicated that the intervention group showed significant learning motivation and class satisfaction [12].

A systematic review was conducted to identify teaching methods of medication administration safety of nursing undergraduates using four electronic databases. The three methods identified were: simulation experience, use of technology and online learning. However, they insisted that those three methods could not be implemented in all the nursing programmes, and the teachers should pay attention to preparing and confirming classroom-based teaching methods. Furthermore, it was suggested to conduct future studies on developing tools considering psychological aspects to assess nursing students’ compliance on medication administration safety [13].

This project has several limitations. The current study used a small convenience sample from a limited target population of nursing students. Therefore, our findings have limited generalizability. Another drawback was that there could be contamination as the post-test was done 8 days later. It should have been done on the same day after teaching and repeated on the 8th day. One of the strengths of the present study was none of the students learned the principles of oral medication administration through a lecture or a practical demonstration before. Therefore, the impact of prior knowledge for the mean knowledge score could be excluded and considered as minimal. As the future implications of this study, it is suggested to assess the skills acquisition and attitude development with regards to the oral medication administration using different teaching methods such as mobile applications, virtual reality simulations and also to conduct a similar study using a larger sample, involving nursing students of other universities and nurses training schools in Sri Lanka.

## Conclusion

This study found that both conventional demonstration method and video-assisted teaching method were equally effective on knowledge acquisition of oral medication administration. Hence, it is recommended that a combination of teaching methods can be adopted to enhance the skill development of the students. A live demonstration is a traditional and preferred method, but a video can also be used. Future studies can be extended to other nursing procedures with the participation of other nursing faculties for uplift the arena of nursing education.

## Supplementary Information

**Additional file 1.** Questionnaire on “Student Nurses” Knowledge Acquisition on Oral Medication Administration.

## Data Availability

The datasets generated and/or analysed during the current study are not publicly available as this study protects the participants’ privacy but are available from the corresponding author on reasonable request.

## Abbreviations

FAHS: Faculty of Allied Health Sciences
SBA: Single Best choice Answer questions
UGC: University Grants Commission
