# Supplementary material for: Student nurses’ knowledge acquisition on oral medication administration: comparison of lecture demonstration vs. video demonstration
Source: BMC Nurs. 2021 Jan 6;20:9. doi: 10.1186/s12912-020-00527-6 (PMC7788856; doi:10.1186/s12912-020-00527-6)
Supplement: Supplementary file 1 — Additional file 1. Questionnaire on “Student Nurses” Knowledge Acquisition on Oral Medication Administration. [file 12912_2020_527_MOESM1_ESM.docx]

Serial Number:……………………

**Questionnaire on “Student Nurses' Knowledge Acquisition on Oral Medication Administration: Comparison of Lecture Demonstration Vs. Video Demonstration”**

You may have been already informed that a study on “Student Nurses' Knowledge Acquisition on Oral Medication Administration: Comparison of Lecture Demonstration Vs. Video Demonstration”. Data will be analyzed only in anonymity. Your participation is voluntary and it will take about 30 minutes to fill the questionnaire.

**Part I – Socio-demographic Data**

*Please tick* (√) *the most appropriate response.*

1. Gender: Male Female
2. Please write your AGE: ……..
3. Nationality :
4. Sinhala
5. Tamil
6. Muslim
7. Other
8. Religion :
   1. Buddhist
   2. Hindu
   3. Christian
   4. Islam
   5. Other
9. Place of residence:
10. Home
11. University Hostel
12. Boarding House

**Part II**

*Please circle the most appropriate response.*

**Q1**. All of the following are required sources to confirm the patient’s identity before administering medication except:

1. Patient’s identification bracelet
2. Patient’s room or bed number
3. Ask the patient
4. Patient’s chart

**Q2.** What should the nurse do first when preparing medications in order to administer to a patient?

A. Check the medication expiration date

B. Check the medication administration record

C. Check the patient’s name

D. Call the pharmacy for administration instructions

**Q3.** What are the six rights of medication administration?

A. Right medication, Right route, Right patient, Right documentation, Right dose, Right time

B. Right patient, Right medication, Right time, Right prescription, Right date, Right documentation

C. Right dose, Right route, Right date, Right assessment, Right documentation, Right medication

D. Right dose, Right patient, Right formulation, Right time, Right documentation, Right medication

**Q4.** The nurse has to take a drug from a container by pouring the medication into

A. the nurse’s hand

B. the container lid

C. the medication administration cup

D. the patient’s hand

**Q5**. The nurse is performing the **second check** when administering oral medications:

A. at the patient’s bedside

B. before the medication is taking out of the bottle or removing the strip

C. when checking the medication order

D. when selecting medications from the medication trolley/ cupboard

**Q6.** What is not an appropriate action when a patient, who is legally responsible for their care, refuses a medication?

A. Notify the ordering physician of patient's refusal to take medication

B. Document the patient's refusal to take medication and the education that you provided

C. Explain the consequences for not taking the medication

D. Force the patient to take it anyway

**Q7.** The nurse should not give oral medications when patient is,

A. having diarrhoea

B. allergy to penicillin

C. unconscious

D. having fractured leg

**Q8.** An elderly patient is scheduled to take five medications each morning. The nurse administering these medications knows to do which of the following?

A. Allow extra time to administer all of the medications

B. Allow the patient to take only the medications she can swallow

C. Crush all the medications before giving them

D. Leave the medications at the bed side so, the patient can take them slowly

**Q9.** Sublingual drug is administered by placing the drug in what part of the body?

A. Between the cheek and upper jaw

B. Under the tongue

C. In the nose

D. In the eyes

**Q10.** When do you document on the medication chart that a medication was administered?

A. Before you give it

B. Before the patient swallows it

C. Immediately after administration

D. within 05 minutes after administration

**Q11.** Before the nurse administers a liquid medication to an 83years old female patient, the nurse should,

A. assess the swallowing reflex by offering sip of water.

B. ask the patient if she would prefer to give the medications to herself.

C. mix thoroughly in a juice.

D. assess the ability to understand information relative to the drug.

**Q12.** The nurse receives an order to give **vitamin D 10mcg bd**. The nurse recognizes it as,

A. Vitamin D 10 micrograms three times per day

B. Vitamin D 10 micrograms two times per day

C. Vitamin D 10 milligrams three times per day

D. Vitamin D 10 milligrams two times per day

**Q13.** Which of the following is the most important factor to check before administering any medications?

1. Antibody levels
2. History of allergies
3. Identified antigen
4. Gender of the patient

**Q14.** Which of the following is true of oral medication administration?

1. It is expensive.
2. It is more difficult to administer.
3. The drug is absorbed through the mouth.
4. The drug is absorbed through the lining of the stomach.

**Q15.** Amount of drug prescribed for a patient by a physician in a given amount of time or at a given frequency is called as the

1. route.
2. number of tablets.
3. dosage.
4. prescription.

**Part III**

*Please put a tick (√) in the relevant box.*

| **Serial No.** | **Statement** | **Strongly Agree** | **Agree** | **Neutral** | **Disagree** | **Strongly Disagree** |
| --- | --- | --- | --- | --- | --- | --- |
| **A1** | I enjoy learning skills through video |  |  |  |  |  |
| **A2** | I enjoy learning skills through a lecture in the lecture theatre |  |  |  |  |  |
| **A3** | I find the skills classes useful |  |  |  |  |  |
| **A4** | I enjoy learning skills through demonstration by a lecturer in the skills lab |  |  |  |  |  |
| **A5** | I felt prepared for the skills class after I watched the videos |  |  |  |  |  |
| **A6** | I feel the videos and skills classes have prepared me for the skills in clinical practice |  |  |  |  |  |
| **A7** | I would like videos to be used more in skills teaching |  |  |  |  |  |
| **A8** | I feel I will use the videos to revise clinical skills in the future |  |  |  |  |  |
| **A9** | I would like to have more demonstration of the skills by the lecturers in skills classes |  |  |  |  |  |
| **A10** | I would prefer if we watched the videos in the skills class |  |  |  |  |  |
| **A11** | I feel motivated to learn the skills through video |  |  |  |  |  |
| **A12** | I am satisfied with teaching oral medication administration by video demonstration |  |  |  |  |  |

-Thank you-
